# Acute and Delayed Effects of Stress Eliciting Post-Traumatic Stress-Like Disorder Differentially Alters Fecal Microbiota Composition in a Male Mouse Model

**DOI:** 10.3389/fcimb.2022.810815

**Published:** 2022-03-01

**Authors:** Allison Hoke, Nabarun Chakraborty, Aarti Gautam, Rasha Hammamieh, Marti Jett

**Affiliations:** ^1^ Oak Ridge Institute for Science and Education (ORISE), Oak Ridge, TN, United States; ^2^ Medical Readiness Systems Biology Branch, Center for Military Psychiatry and Neuroscience Research (CMPN), Walter Reed Army Institute of Research (WRAIR), Silver Spring, MD, United States

**Keywords:** PTSD, C57BL/6J, stress, social defeat, microbiome

## Abstract

The association between the shift in fecal resident microbiome and social conflicts with long-term consequences on psychological plasticity, such as the development of post-traumatic stress disorder (PTSD), is yet to be comprehended. We developed an aggressor-exposed (Agg-E) social stress (SS) mouse model to mimic warzone-like conflicts, where random life-threatening interactions took place between naïve intruder mice and aggressive resident mice. Gradually these Agg-E mice developed distinct characteristics simulating PTSD-like aspects, whereas the control mice not exposed to Agg-E SS demonstrated distinct phenotypes. To further investigate the role of Agg-E SS on the resident microbiome, 16S rRNA gene sequencing was assayed using fecal samples collected at pre-, during, and post-SS time points. A time agonist shift in the fecal microbial composition of Agg-E mice in contrast to its controls suggested a persistent impact of Agg-E SS on resident microbiota. At the taxonomic level, Agg-E SS caused a significant shift in the time-resolved ratios of *Firmicutes* and *Bacteroidetes* abundance. Furthermore, Agg-E SS caused diverging shifts in the relative abundances of *Verrucomicrobia* and *Actinobacteria*. An *in silico* estimation of genomic potential identified a potentially perturbed cluster of bioenergetic networks, which became increasingly enriched with time since the termination of Agg-E SS. Supported by a growing number of studies, our results indicated the roles of the microbiome in a wide range of phenotypes that could mimic the comorbidities of PTSD, which would be directly influenced by energy deficiency. Together, the present work suggested the fecal microbiome as a potential tool to manage long-term effects of social conflicts, including the management of PTSD.

## 1 Introduction

There is increased interest in the gut-brain axis and the potential role of the gut microbiome in regulating the mental health of the host (Clapp et al., 2017; Dinan and Cryan, 2017; Osadchiy et al., 2019). Studies suggested a bidirectional relationship between the host and the microbiome (Clapp et al., 2017; Dinan and Cryan, 2017; Osadchiy et al., 2019), and understanding and modeling of this relationship could result in diagnostic markers of the host stress response, and thus a way to identify precision treatments. Communication along the gut microbiota-brain access consists of various routes including the immune system, the endocrine hypothalamic-pituitary adrenal axis, and the autonomic and enteric nervous system, using metabolites such as short-chain fatty acids, tryptophan metabolites, and secondary bile acids (Wikoff et al., 2009; Tolhurst et al., 2012; Yano et al., 2015; Cryan et al., 2019). The alteration of the composition of the gut microbiome is potentially associated with pathophysiology of diseased states, including autism, anxiety, stress, major depressive disorder, schizophrenia, obesity, irritable bowel syndrome, and bipolar disorder (Cryan et al., 2019).

The connection between the gut microbiome and brain functions is still a relatively new field, and studies are ongoing to give understanding into this relationship. There is yet considerable speculation as to whether changes in the microbiota are fundamental to the pathophysiology of neurological and psychiatric disorders (Clapp et al., 2017; Dinan and Cryan, 2017; Cryan et al., 2019), and it is beneficial to gain insight into the potential relationships. In the current study, we are focusing on post-traumatic stress disorder (PTSD), which is a condition of persistent mental and emotional stress that is typically triggered by experiencing life-threatening events (Battle, 2013). PTSD has persistent symptoms that include re-experiencing of the traumatic event (flashbacks), avoidance of stimuli associated with the trauma, numbing, significant distress, and social impairment (Battle, 2013; Auxemery, 2018). The symptoms of PTSD closely align and significantly overlap with co-morbidities, so the pathophysiology of PTSD remains poorly understood, and lacks objective diagnoses and robust treatments (Auxemery, 2018; Compean, 2018). The number and severity of additional stressful life events signal a higher risk to develop PTSD (Maes et al., 2001). To study the gut microbiome and its role and function in disease, murine models remain the most popular choice (Nguyen et al., 2015; Schoner et al., 2017). The murine model has the advantages of allowing investigations of gut microbiome disruptions in a controlled environment, permits examinations and interventions not possible in humans, and the mouse model itself includes well-known genetics with a homogenous genetic background, low cost of maintenance, short life cycle, and the gut anatomy and physiology shares similarities to humans (Nguyen et al., 2015). Limitations of the murine model include that despite principal similarities, the mouse model cannot fully capture human systems, the interaction between the gut and the host is host-specific, thus using a mouse model may not be as applicable in humans, and the inbred mouse strains does not capture the genetic variations found the human populations (Nguyen et al., 2015).

In the present study, we probed a previously validated C57BL/6J mouse model simulating PTSD-like features (Hammamieh et al., 2012; Chakraborty et al., 2015; Gautam et al., 2015; Muhie et al., 2015; Muhie et al., 2017; Gautam et al., 2018). This model was developed to mimic warzone-like conflicts, where random life-threatening interactions took place between naïve intruder and aggressive resident mice. The model was modified from a social stress (SS) model of traumatic stress, which involves mice being stressed by exposures to trained aggressor mice for 6-hour sessions daily for ten days in a “cage-within-cage” resident-intruder protocol. During the 6-hour session, the aggressor-exposed (Agg-E) mouse was removed from the smaller cage for up to three random times and exposed to the trained aggressor mouse for one minute or ten strikes, whichever came first. By the end of the ten days of SS, the Agg-E SS mice experienced the effects of acute stress, including gains in body weight, increased body temperature, metabolite and transcriptomic changes, cardiac inflammation and cardiomyopathy, alterations in brain structure, liver inflammation, and behaviors indicative of fear and anxiety (Hammamieh et al., 2012; Chakraborty et al., 2015; Gautam et al., 2015). There was evidence in the brain of Agg-E SS mice of increased activations of pathways related to anxiety, mood disorders, impaired cognition, with enrichment in signaling pathways associated with PTSD-comorbid conditions, and inhibition of processes connected with synaptic plasticity and neurogenesis (Muhie et al., 2015; Muhie et al., 2017). Furthermore, metabolic dysfunction is also a known comorbidity of PTSD (Michopoulos et al., 2016; Mellon et al., 2018), and our previous study found that molecular changes that were associated with PTSD-comorbidities were also significantly associated with the differentially regulated genes common among Agg-E SS mice in the blood, hemibrain, and spleen (Muhie et al., 2017). The effects of acute stress seen in the Agg-E SS mice are also evident in humans experiencing PTSD, including gains in body weight (Mitchell et al., 2016), alterations in metabolites (Mellon et al., 2019) and transcriptomics (Segman et al., 2005; Zieker et al., 2007; Yehuda et al., 2009; Pitman et al., 2012), and there is an increased risk of cardiovascular disease in patients with PTSD (Pitman et al., 2012; Celano et al., 2016; Edmondson D, 2017). Furthermore, humans with PTSD experience alterations in brain structure, such as a lower volumes in the hippocampus and ventromedial prefrontal cortex (Pitman et al., 2012), and chronic liver disease which is often coupled with alcohol dependence thus exacerbating the problem (Samala et al., 2018; Forehand et al., 2019). The increased body temperature observed in the Agg-E SS mice has also been seen in other social defeat models, suggestive of the chronic stress (Koolhaas et al., 1997; Meerlo et al., 1997; Keeney et al., 2001).

Our previous microbiome study using the Agg-E SS model examined the acute changes to the gut microbiome over the ten-day Agg-E SS (Gautam et al., 2018), and the current study utilized a more longitudinal approach also encompassing a pre-stress control and post-stress time points, as we collected fecal samples from control and Agg-E mice before SS, during SS, and one and four weeks after the termination of SS. The current study also aimed to capture that the change in the microbiome, as evidenced by SS, had the potential to cause increased intestinal permeability. Stress affects the gastrointestinal tract in numerous ways, but its effect on intestinal barrier function is mainly through increased permeability allowing the movement of harmful microorganisms, pro-inflammatory factors and antigens (Farhadi et al., 2003; Camilleri, 2019). Animal models of disease have documented a three point relationship consisting of a disease phenotype, intestinal barrier change, and altered microbiota, but the directionality of the relationship as it applies to most diseases is not clear as to what may be the cause and what is the effect, and most human models do not investigate all three in tandem (Camilleri, 2019).

In this longitudinal study, we assessed the 16S ribosomal RNA gene in fecal samples to determine the gut microbiota composition in the presence of and during recovery from SS, in an effort to gain valuable insight into characterizing the role of the microbiome in PTSD and to work towards developing therapeutic strategies. The objective of this study was to understand the association between PTSD pathophysiology and the long-term shift in microbiota. The ultimate objective is to suggest potential mitigation strategies or nutraceutical interventions to restore the pre-stress condition of microbiota and thereby minimize the risk of PTSD prevalence.

## 2 Materials and Methods

### 2.1 Mice

All animal experiments were approved in writing by the Institutional Animal Care and Use Committee of the United States Army Medical Research and Materiel Command and the United States Army Center for Environmental Research and were conducted in compliance with the Animal Welfare Act and other Federal statutes and regulations relating to animals and experiments involving animals, adhering to principles stated in the Guide for the Care and Use of Laboratory Animals (Council, 2011) in facilities fully accredited by AAALAC International. All mice were purchased from Jackson Laboratory, Bar Harbor, ME, USA. All mice had ad libitum access to food and water and were kept in a temperature-controlled room (21 ± 2°C) on reverse 12/12-hour light/dark cycle (lights on at 06:00 PM and off at 06:00 AM). Mice were randomly assigned to aggressor-exposed (Agg-E) and cage-control groups (N=5 per group). The aggressor mice were SJL albino male mice (5 to 6 weeks old, weighing 30-35g when purchased) and were individually housed in polycarbonate cages (48 x 27 x 20 cm). Male mice were chosen as aggressors since they tend to be aggressive over a wide range of conditions (Miczek et al., 2001), whereas female mice are minimally aggressive when subjected to the isolation housing in this protocol, outside of periods of pregnancy or suckling (Newman et al., 2019). Aggressor mice were trained to be hostile to the intruder following a protocol described earlier (Hammamieh et al., 2012). Briefly, the aggressor mice were individually housed for 1 month prior to the experiment to induce aggressiveness and territorial behavior due to isolation and were trained to assault intruders to their home cage, and their behavior was monitored. Those not meeting expectations of aggressiveness were not included in the study. The control and subject mice were male C57BL/6J mice (5 to 6 weeks old, weighing 20-25g) and were individually housed in a different room from the aggressor mice for one week prior to and during the experiment under the same environmental conditions.

### 2.2 Aggressor Exposure

As done in our previous study, we used a modified “cage-within-cage” resident-intruder protocol where Agg-E C57BL/6J mice were placed in a wire mesh cage (17.5 x 14 x 7.5 cm) inside an aggressors large plastic home cage for 6-hours a day for ten consecutive days without access to food or water (Hammamieh et al., 2012). Briefly, during the six-hour “cage-within-cage,” Agg-E mice were randomly placed in physical contact with the aggressor mouse, at random intervals, for 1 min or 10 strikes, whichever came first. On average, the Agg-E SS mice received 10 strikes in 48 seconds during Agg-E (Hammamieh et al., 2012), and the aggressor mice were rotated through the Agg-E SS mice, to account for any variability in aggressiveness. We previously validated the effectiveness of this social defeat model and performed ethogram evaluations using a 5 min partition test at 1 day, 4 weeks, and 6 weeks following the 10-day Agg-E to evaluate behavioral patterns in the control versus Agg-E SS mice (Hammamieh et al., 2012), to ensure the Agg-E SS mice developed PTSD-like features.

### 2.3 Sample Collection

**Figure 1:**
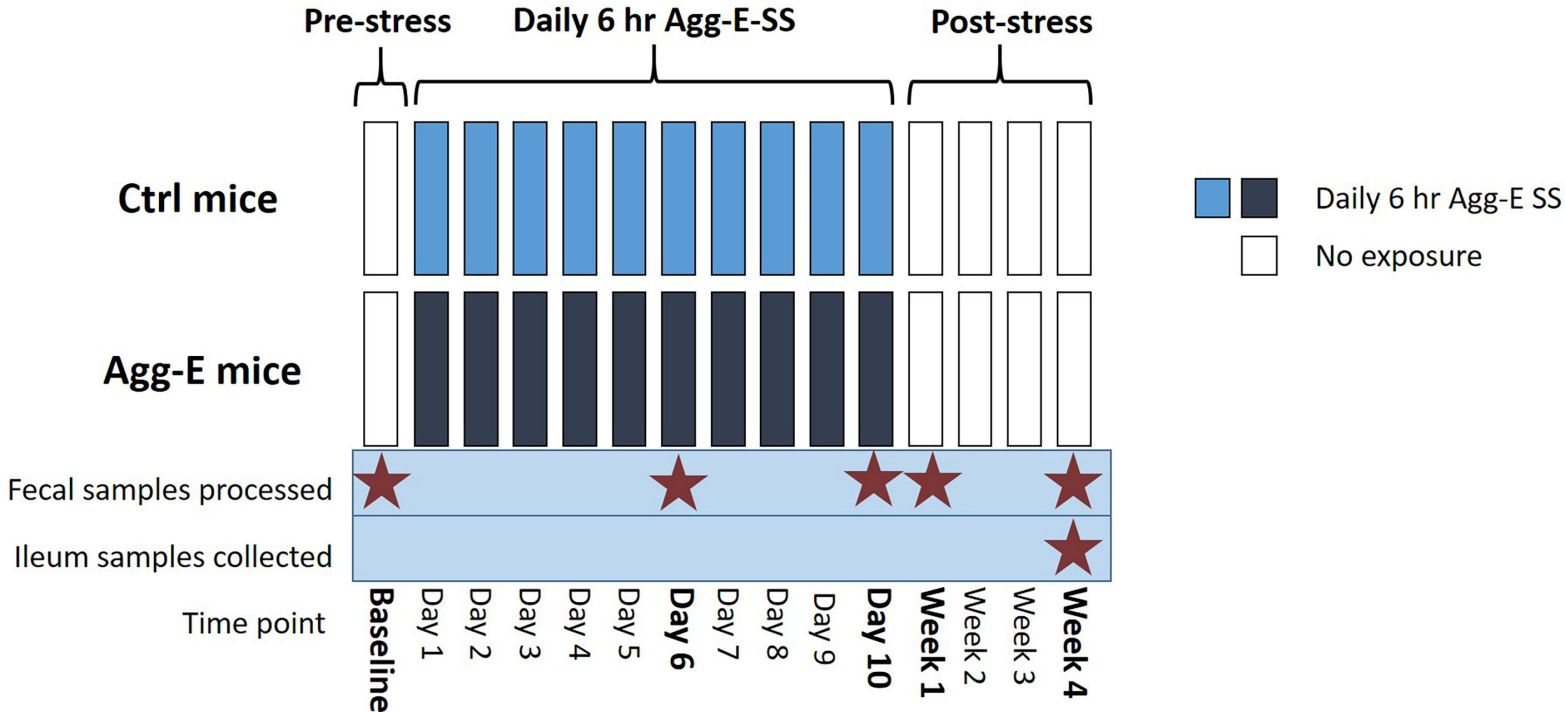
Overall Experimental Strategy. Agg-E SS C57BL/6J male mice were exposed to trained aggressor SJL mice for 6-hours/day in a “cage-within-cage” model. Fecal pellets were collected at Baseline, Day 6 and Day 10 during the Agg-E SS, and at Week 1 and Week 4 post-SS from Agg-E SS and control mice, and stored at -80°C. Ileum samples were collected at Week 4 post-SS. (Ctrl, control; Agg-E, aggressor-exposed; SS, social stress; light blue, daily 6-hour/day Agg-E SS; dark blue, control mice undergoing “cage-within-cage” 6-hour/day but no Agg-E SS; white, no “cage-within-cage” or Agg-E SS).

Fecal pellet samples were collected from mice before the “cage-within-cage session” each morning when the mice were being weighed to minimize handling stress. The time points the fecal pellets were collected were during social stress, and in the morning for the Baseline, Week 1, and Week 4 post-SS samples (**Figure 1**). The fecal pellets were stored at -80°C until DNA extraction. Mice were euthanized by cervical dislocation, alternating between Agg-E and control mice to control for time of day effects. The ilea were removed, digesta was flushed out by rinsing with sterile saline, and the tissues were flash frozen in liquid nitrogen and stored at -80° C until DNA extraction.

### 2.4 Nucleic Acid Extraction

The fecal samples were kept continuously frozen until extraction, and the DNA was isolated from the fecal samples using the DNeasy PowerSoil kit (QIAGEN, Inc., Hilden, Germany) according to the manufacturer’s protocol. The extracted fecal DNA was used for 16S rRNA sequencing and taxa validation qPCR.

The ilea from the mice was homogenized using the Precellys Evolution (Bertin Instruments, France) in TRIzol Reagent (Thermo Fisher Scientific, Waltham, MA), the equivalent of 30mg of tissue in TRIzol Reagent was further homogenized, used for RNA extraction using the RNeasy Mini Kit (QIAGEN, Inc.) following the manufacturer’s protocol, and the RNA was used for qPCR.

### 2.5 Library Preparation and Sequencing

We followed the Illumina 16S Metagenomics Library Preparation manual (Illumina, Inc., San Diego, CA) according to the manufacturer’s protocol. Briefly, we used previously designed primers to isolate the hyper-variable V3 and V4 region of the 16S rRNA amplicon (Klindworth et al., 2013), the samples were barcoded using Nextera indexes, and an amplicon of approximately 460 bp was generated. The libraries were pooled and sequenced on the Illumina MiSeq platform, using paired-end 300 bp reads and Illumina MiSeq v3 reagents. The end of each read was overlapped to generate high quality, full-length reads of the V3 and V4 regions.

### 2.6 Quantitative Polymerase Chain Reaction (qPCR)

#### 2.6.1 Intestinal Permeability qPCR

A literature search identified intestinal permeability genes of interest, and the genes of *muc2*, *muc3*, and *muc5b* (Gaudier et al., 2004), *tlr1*, *tlr2*, and *tlr4* (Sapone et al., 2011), *mmp9* (van Horssen et al., 2006), *nfĸb* and *tnf-α* (Ma et al., 2004), as well as the housekeeping genes of *gapdh*, *actb*, and *ubc* were selected. Primers were designed for the genes using the PrimerQuest Tool (IDT Integrated DNA Technologies, Coralville, IA) (**Supplemental Table S1**), and the extracted ileal RNA was used with the RT^2^ First Strand Kit (QIAGEN) for cDNA synthesis, followed by qPCR using the RT² SYBR Green qPCR Mastermix (QIAGEN) on the QuantStudio 7 Flex Real-Time PCR System (ThermoFisher Scientific), following the cycling conditions of 95°C for 10 min followed by 40 cycles of 95°C for 15 sec and 60°C for 1 min, with data acquisition at every cycle.

#### 2.6.2 Taxa Validation qPCR

For *Bifidobacterium pseudolongum*, a literature search was performed, and primers were identified for *Bifidobacterium pseudolongum*, as well as the subspecies *pseudolongum* and *globosum* (**Supplemental Table S1**) (Lugli et al., 2019). The standards of *Bifidobacterium pseudolongum subsp. pseudolongum* Mitsuoka (ATCC^®^ 25526) and *Bifidobacterium pseudolongum subsp. globosum* (ATCC^®^ 25865) were reconstituted in 1xPBS (Gibco, ThermoFisher Scientific) and extracted using the DNEasy UltraClean Microbial Kit (Qiagen, Inc.) according to the manufacturer’s protocol. The extracted DNA was used as standards and a dilution series was run with the samples on qPCR to determine the concentrations of *B. pseudolongum* in the fecal samples. The qPCR was done using the RT² SYBR Green qPCR Mastermix on the QuantStudio 7 Flex Real-Time PCR System, following the cycling conditions of 95°C for 10 min followed by 40 cycles of 95°C for 15 sec and 60°C for 1 min, with data acquisition at every cycle.

The validation qPCR for *Akkermansia muciniphila* in the fecal samples over the time course followed the protocol for RT-PCR using the Microbial DNA qPCR Assay Kit (QIAGEN, Inc). Briefly, 5 ng of DNA was used for the assay which included: extracted genomic DNA, Microbial DNA Positive Control, Negative Template Control, and a PCR Positive Control. The qPCR was performed with the QuantStudio 7 Flex Real-Time PCR System, following the cycling conditions of 95°C for 10 min followed by 40 cycles of 95°C for 15 sec and 60°C for 2 min, with data acquisition at every cycle. An *Akkermansia muciniphila* standard (ATCC, Manassas, VA, USA, Cat#BAA835D-5) dilution series was prepared and run with the qPCR to determine the concentrations of *A. muciniphila* in the fecal samples.

### 2.7 Data Analysis

The initial data quality assessment, processing and chimera detection on the sequencing reads were processed using QIIME2 v.2019.4 (Bolyen et al., 2019) following a standard procedure on demultiplexed sequences. Raw sequence reads were joined using the join-pairs method with the q2-vsearch plugin (Rognes et al., 2016) followed by initial quality filtering based on quality scores and denoising with Deblur (Amir et al., 2017) with a trim length of 438 nt. All amplicon sequence variants (ASVs) were aligned with mafft (Katoh et al., 2002) (*via* q2‐alignment) and used to construct a phylogeny with fasttree2 (Price et al., 2010) (*via* q2‐phylogeny). Alpha-diversity metrics [Chao1 (Chao, 1984), Shannon (Shannon and Weaver, 1949), Faith’s Phylogenetic Diversity (Faith, 1992), and Simpson indexes (Simpson, 1949)], beta diversity metrics of Weighted UniFrac (Lozupone et al., 2007), and Principle Coordinate Analysis (PCoA) were estimated using q2‐diversity after samples were rarefied (subsampled without replacement) to 2251 sequences per sample. Prism (GraphPad, San Diego, CA) was used to make the alpha diversity plots and to determine alpha group significance using a 2-way analysis of variance (ANOVA), and Tukey’s test was used for multiple comparisons. The Adonis plugin in QIIME2 (Anderson, 2001; Oksanen et al., 2018) was used to run an analysis of variance using distance matrices, which is directly analogous to multivariate analysis of variance (MANOVA) for beta group significance. The plugin q2-longitudinal (Bokulich et al., 2018) was used in QIIME2 to on the alpha and beta diversity metrics to generate the linear mixed effects models and to see the volatility. To assign taxonomy to the ASVs, a Naïve Bayes Classifier was trained on the 16S rRNA V3-V4 region with the specific primers and the GreenGenes v13.8 99% operational taxonomic unit (OTU) database of reference sequences (McDonald et al., 2012) using q2-feature-classifier (Bokulich et al., 2018) *via* classify-sklearn (Pedregosa et al., 2011). The GreenGenes v13.8 database was released in 2013 and does not include the most recent updates to the microbial tree but was chosen because our previous manuscript investigating the microbiome of the social stress mouse model (Gautam et al., 2018) was analyzed using this database. Prism was used for the analysis and plotting temporal taxa data for the control and Agg-E SS mice. Rank abundance profiling at the genus level was performed on the 52 genera identified from QIIME2, and the top 20 genera were ranked for both Agg-E SS and control samples irrespective of time point, using their relative abundance. Rank abundance profiling at the species level was performed on the 57 species identified from QIIME2, and the top 10 species were ranked based on their relative abundance, irrespective of time and SS. The top two identified species were selected for use for taxa validation qPCR: *Bifidobacterium pseudolongum* and *Akkermansia muciniphila.* MetaCyc pathway abundances (Caspi et al., 2014) were predicted based on 16S rRNA sequencing ASVs using Phylogenetic Investigation of Communities by Reconstruction of Unobserved States (PICRUSt2) (Ye and Doak, 2009; Louca and Doebeli, 2018; Barbera et al., 2019; Czech et al., 2020; Douglas et al., 2020). The PICRUSt2 pathways and the sequencing-derived taxonomic ASVs were analyzed using the Bioconductor package DESeq2 (Love et al., 2014) in R (version 4.0.2). The DESeq2 results of significant MetaCyc pathways were fed into Linear discriminant analysis effect size (LEfSe) to generate cladograms; LEfSe was not used for analysis.

The qPCR for intestinal permeability markers were normalized by the housekeeping genes, and fold change of each Agg-E SS sample expression relative to the expression in healthy controls was calculated by the 2^(-ΔΔCt) method (Livak KJ, 2001), and was graphed in Prism. The qPCR for the taxa validation was analyzed and graphed in Prism.

## 3 Results

### 3.1 Taxonomic Changes Due to Social Stress

To explore the longitudinal relationship between SS-induced changes on the microbiome, we characterized the fecal microbiota by sequencing the fecal 16S rRNA from the feces of the control and Agg-E mice over the time course (**Supplemental Figure 1**). The number of sequencing reads and the number of amplicon sequence variants (ASVs) for the samples are outlined in **Table S2** and **Supplemental Figure S1**. The average read count was approximately 166,000 for the raw reads, with 46,000 average reads after initial filtering, processing, and read joining. There was an average of 171 ASVs per sample, and 1490 different ASVs were identified. Around 78% of the ASVs were common between control and Agg-E SS mice. The average Q-score for forward reads was 36 and was 32 for forward and reverse reads.

**Figure 2:**
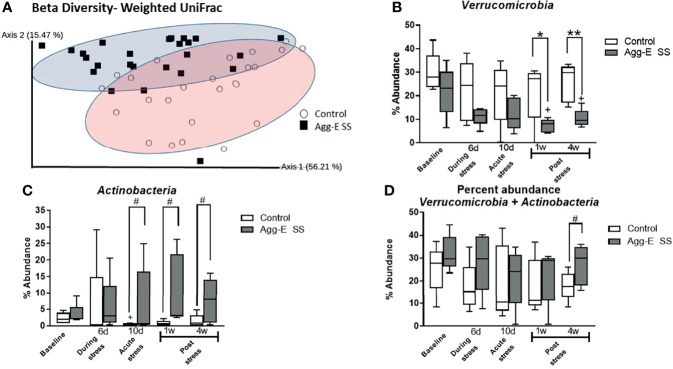
Beta diversity and abundance of phyla. **(A)** Beta diversity metric Weighted UniFrac Principal Coordinate Analysis (PCoA) plot of Control (○) and Agg-E SS (▪) samples. (2-way PERMANOVA: Stress p < 0.001, Time p < 0.05). **(B)** Percent abundance of phylum *Verrucomicrobia*. (2-way ANOVA: Time p = 0.06, Stress p < 0.001) **(C)** Percent abundance of phylum *Actinobacteria*. (2-way ANOVA: Stress p < 0.05). **(D)** Percent abundance of the summation of phyla *Verrucomicrobia* and *Actinobacteria*. (2-way ANOVA: Stress p < 0.05) (Agg-E SS, aggressor-exposed social stress mice, #p-value: 0.1<# < 0.05, *p-value < 0.05, **p-value < 0.01. white bars, control; dark gray bars, Agg-E SS). The whiskers represent the minimum and maximum values.

Following the social stress, the Chao1 alpha diversity within the entire sample set was significantly altered in response to time (2-way ANOVA, p<0.05) (**Supplemental Figure S2A**). In addition, there was an initial decreasing trend in the alpha diversity in the Agg-E SS samples, as highlighted by the decline in the Chao1 index measurement between Baseline and Day 6 (t-test, p = 0.05) during the Agg-E SS. However, no significant differences of SS or time were seen in the alpha diversity metrics of the Shannon diversity index (**Supplemental Figure S2B**) (which places more weight on species richness) Simpson’s Index (**Supplemental Figure S2C**) (which places more weight on species evenness) and Faith’s Phylogenetic Distance (**Supplemental Figure S2D**) (which is a qualitative measure of biodiversity that incorporates phylogenetic difference between species using the sum of branch lengths). The alpha diversity metrics were also explored using the QIIME2 longitudinal plugin, and the linear mixed effects models and volatility plots, respectively, of control and Agg-E SS mice over the time course are shown in **Supplemental Figure S3** for Chao1 (**Supplemental Figures S3A, E**), Shannon’s Index (**Supplemental Figures S3B, F**), and Simpson’s Index (**Supplemental Figures S3C, G**). The volatility plots are used to examine how the diversity changed over time in each subject, and the results indicated that for Chao1, both control and Agg-E SS mice experienced a similar rate of phylogenetic transition, as they both decreased at Day 6, increased at Day 10, then experienced a slow decreasing plateau with decreased variance by Week 4 post-stress (**Supplemental Figure S3E**). The Shannon’s Index (**Supplemental Figure S3F**) in Agg-E SS mice decreased to Day 6, then increased to Day 10, followed by a plateau whereas control mice gradually decreased to Week 1, followed by a slow increase. Simpon’s Index (**Supplemental Figure S3G**) showed little variation between control and Agg-E SS mice, as both experienced a slight decrease during SS, with a convergence of lines by Week 4. Furthermore, we measured beta diversity levels using Weighted UniFrac and visualized the output using Principal Coordinate Analysis (PCoA) (**Figure 2A** and **Supplemental Figure S2E**). The relative abundance profiles for all of the phyla showed 56.21% variance at Principle Component (PC) 1 and 15.47% variance in the PC2 scale. The linear mixed effects model and volatility plots for the Weighted UniFrac are shown in **Supplemental Figures S3D, H**, respectively. The volatility plot decreased in both control and Agg-E SS mice from Day 6 to Day 10, with control mice then decreased to Week 1, then increased whereas Agg-E SS mice increased after Day 6. There was also increased variability following Day 6. The gut microbial communities demonstrated a significant time-independent difference between the control and Agg-E SS samples revealing shorter distances between intra-group samples than between group samples. A multi-factor Adonis test (an analysis of variance using distance matrices) showed the clear separation between control and Agg-E SS mice on the basis of social stress (p = 0.001) and also on time (p<0.05). The interaction between time and stress emerged insignificant.

The rank abundance profile of the bacterial taxa of the Agg-E SS and control samples, respectively, were dominated across all time points by the phyla *Firmicutes* (43 and 49%, respectively) and *Bacteroidetes* (30 and 30%, respectively), followed by *Verrucomicrobia* (24 and 13%, respectively), *Actinobacteria* (2 and 7%, respectively), and *Proteobacteria* (0.7 and 0.9%, respectively) across the time points (**Supplemental Figure S4**). Most phylum relative abundances were comparable between control and Agg-E SS mice, but the percent relative abundance of the phyla *Verrucomicrobia* and *Actinobacteria* (2-way ANOVA, p <0.001 and p <0.05, respectively) were both significantly correlated to SS over the time course, although the interaction of stress and time was not significant (**Figures 2B–D**). In the phylum *Verrucomicrobia*, there was significant differences between the control and Agg-E SS mice at Week 1 (p <0.05) and Week 4 (p = 0.005). The change in the elevations of slopes of the linear regression lines between control and Agg-E SS mice for *Verrucomicrobia* (**Figure 2B**, slopes: F = 1.053, p = 0.3345, elevations or intercept: F = 20.38, p = 0.0027) and *Actinobaceria* (**Figure 2C**, slopes: F = 4.591, p = 0.0759, elevations or intercept: F = 8.556, p = 0.0222) were significant. In the phylum *Verrucomicrobia*, the change elevations in slopes in the linear regression lines between control and Agg-E mice from acute (Day 10) to post-stress (Week 1 and Week 4) were significant (slopes: F = 1.530, p = 0.3416, elevation or intercepts: F = 31.19, p = 0.0113). In the phylum *Actinobacteria*, the change in elevation of slopes in the linear regression lines between control and Agg-E mice from acute (Day 10) to post-stress (Week 1 and Week 4) was significant (slopes: F = 0.01227, p = 0.9219, elevation or intercepts: F = 35.60, p = 0.0094). When comparing the sum of the percent abundance of *Verrucomicrobia* and *Actinobacteria*, SS was significant (p<0.05), and the change in slopes between Control and Agg-E mice was significant (slopes: F = 0.006262, p = 0.9395, elevation or intercept: F = 10.33, p = 0.0148) (**Figure 2D**). The taxonomic data also showed that the ratio of the phyla *Firmicutes* (**Supplemental Figure S5A**) and *Bacteroidetes* (**Supplemental Figure S5B**) individually did not show the impact of time or social stress, but was still vulnerable to PTSD-eliciting stress, as the *Firmicutes*/*Bacteroidetes* Log_10_ ratio (**Supplemental Figure S5C**) was impacted by SS and time (2-way ANOVA, p < 0.001 and p<0.05, respectively). The ratio initially increased from Baseline to Day 6, where it remained steady until it decreased closer to the Baseline values by Week 4 post-stress.

**Figure 3:**
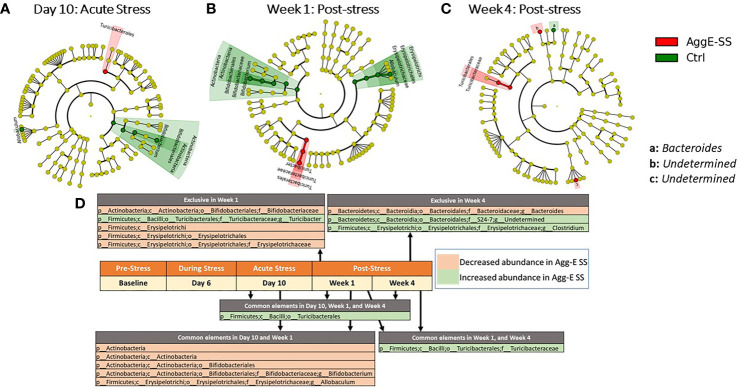
Significant taxonomy cladograms, Agg-E SS *vs* Control, normalized by baseline. Deseq2 analysis of taxonomic ASVs were fed into Linear discriminant analysis effect size (LEfSe) to generate cladograms to graphically represent the significant taxa in their taxonomic tree; LEfSe was not used for analysis. **(A)** Day 10: Acute Stress. **(B)** Week 1: Post-stress. **(C)** Week 4: Post-stress. **(D)** Significant taxa at time points for Agg-E SS versus Control mice, normalized by baseline from Deseq2 (padj < 0.1). Taxa exclusive to a particular time point are listed above the center timeline, whereas taxa in common between time points are listed below the timeline. (light red, decreased abundance in Agg-E SS; light green, increased abundance in Agg-E SS; Agg-E SS, aggressor-exposed social stress; Ctrl, control).

There were significant taxonomic differences between Agg-E SS mice and control mice at Day 10 of SS (**Figure 3A**), Week 1 (**Figure 3B**), and Week 4 (**Figure 3C**). Furthermore, the abundance of order *Turcibacterales* was increased in Agg-E SS mice at Day 10, and Week 1 and Week 4 of post-stress, and family *Turicibacteraceae* was increased in Agg-E SS mice at Week 1 and Week 4 (**Figure 3D** and **Supplemental Table S3**). In Day 10 and Week 1 post-SS, the phylum *Actinobacteria*, class *Actinobacteria*, order *Bifidobacteriales*, and genera of *Bifidobacterium* and *Allobaculum* were decreased in Agg-E SS mice. Additionally, specific taxa were impacted exclusively at a particular post-stress time point, as at Week 1 the genus *Turicibacter* was increased in abundance in Agg-E SS, and families *Bifidobacteriaceae* and *Erysipelotrichaceae*, the class *Erysipelotrichi*, and the order *Erysipelotrichales* were decreased in Agg-E SS mice (**Figure 3D**). At Week 4 post-stress, the genus *Clostridium* and an undetermined genus of the family *S24-7* were increased in Agg-E SS, whereas the genus *Bacteroides* was decreased in Agg-E SS (**Figure 3D**). The specific details for the Deseq2 results for the taxonomy are provided in **Supplemental Table S3**. When considering time only, one taxa emerged as significant, the phylum *Firmicutes*, which had decreased abundance in the Agg-E SS mice (**Supplemental Table S4**).

Rank abundance profiling at the genus level was performed (**Supplemental Table S5**), and the control mice had 15 of the top 20 genera (out of 52 identified genera) originated from the phylum *Firmicutes*, and the top two genera were *Lactobacillus* and *Bacteroides*. The Agg-E SS mice had 14 of the top 20 genera originated from *Firmicutes*, and the top two genera were comprised of *Akkermansia* and *Lactobacillus*. We selected a few of the most abundant species identified by sequencing (**Supplemental Table S6**), which consisted of *Bifidobacterium pseudolongum* and *Akkermansia muciniphila*, and conducted real-time PCR using primers corresponding to the species and bacterial DNA as a standard control. For *B. pesudolongum*, we compared the number of reads of the sequencing data (**Supplemental Figure S6A**) to the RT-PCR data for Agg-E SS and control mice for the subspecies *B. pesudolongum* subspecies *pseudolongum* (**Supplemental Figure S6B**) and *B. pesudolongum* subspecies *globsum* (**Supplemental Figure S6C**) and found a very similar trend, corroborating the sequencing data. *B. pesudolongum* abundance in control mice increases to Week 1, and then decreases, whereas it remains relatively steady in abundance throughout the time course in the Agg-E SS mice. Furthermore, we compared the observed sequencing data from *Akkermansia muciniphila* (**Supplemental Figure S7A**) to the RT-PCR data (**Supplemental Figure S7B**) and found a similar trend of an abundance of *A. muciniphila* in the Agg-E SS and control mice over the time course. The abundance of *A. muciniphila* decreased from Baseline to Day 6, then increased to Week 1 and then decreased again at Week 4 in the sequencing reads. For the qPCR data in the control mice, *A. muciniphila* decreased from Baseline to Day 6, and then continued steadily until it increased at Week 4. Whereas, in the Agg-E SS mice, *A. muciniphila* increased from Baseline to Day 6, then remained steadily through Week 4.

### 3.2 Pathway Analysis

**Figure 4:**
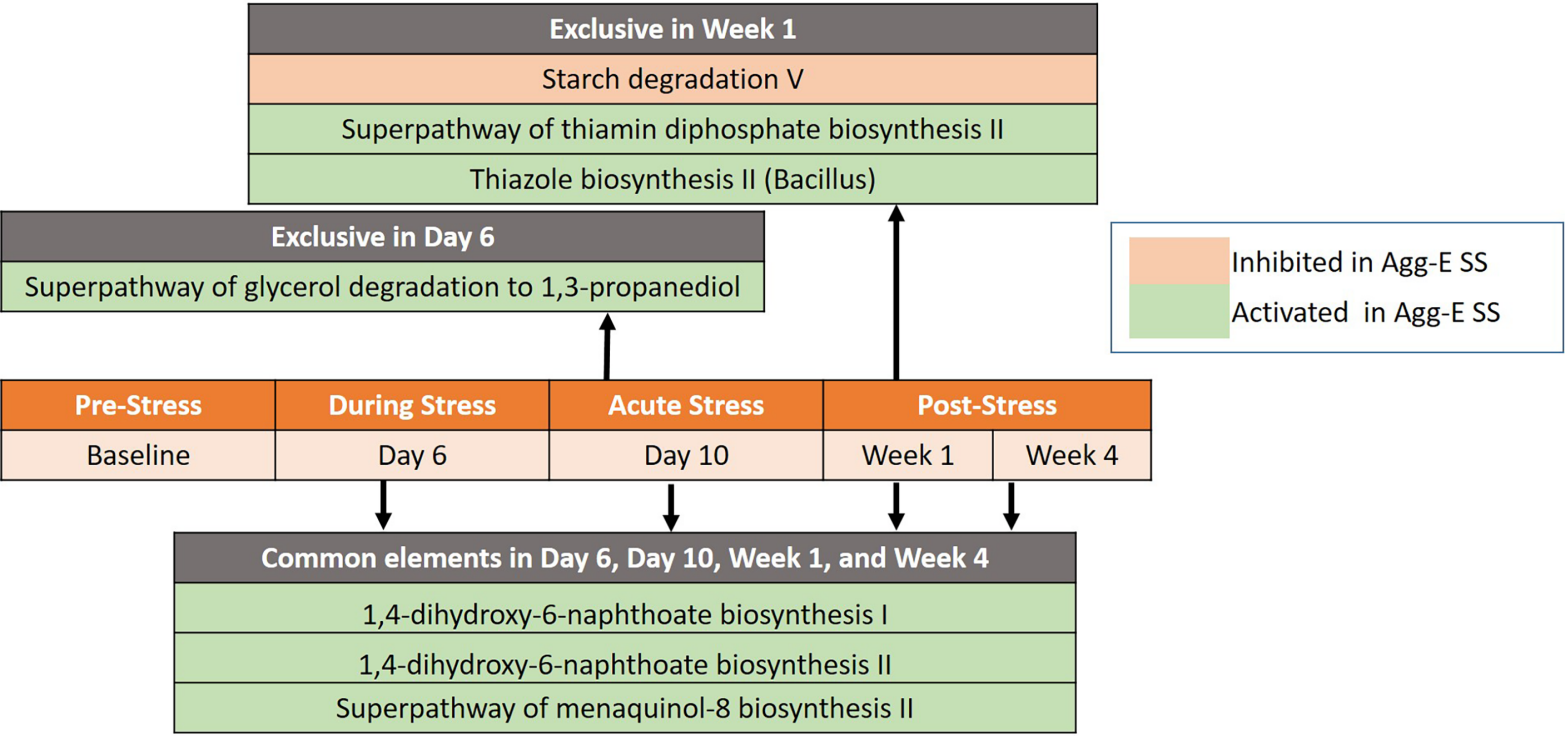
Significant pathways between Agg-E SS *vs* Control mice, normalized by baseline from Deseq2 (padj < 0.1). Those pathways exclusive to a particular time point are listed above the center timeline, whereas pathways in common between time points are listed below the timeline (light red, inhibited in Agg-E SS; light green, activated in Agg-E SS; Agg-E SS, aggressor-exposed social stress).

Seven differentially regulated pathways were detected out of 267 identified pathways (Deseq2, padj < 0.1) after correcting for baseline and time (**Figure 4** and **Supplemental Table S7**). The time points of Day 6, Day 10, Week 1, and Week 4 had three pathways in common that were activated in Agg-E SS mice, consisting of 1,4-dihydroxy-6-naphthoate biosynthesis I, 1,4-dihydroxy-6-naphthoate biosynthesis II, and the Superpathway of menaquinol-8 biosynthesis II. Day 6 of acute stress had an activation in Agg-E SS mice of the Superpathway of glycerol degradation to 1,3-propanediol. Week 1 of post-SS had an activation in Agg-E SS mice of the Thiazole biosynthesis II (*Bacillus*) and the Superpathway of thiamin diphosphate biosynthesis II, and had an inhibition of Starch degradation V. There were a number of differentially regulated time-dependent pathways corrected for Baseline (**Supplemental Table S8**), with 36 pathways being inhibited in Agg-E SS mice and 31 pathways being increased in Agg-E SS mice.

### 3.3 Intestinal Permeability

**Figure 5:**
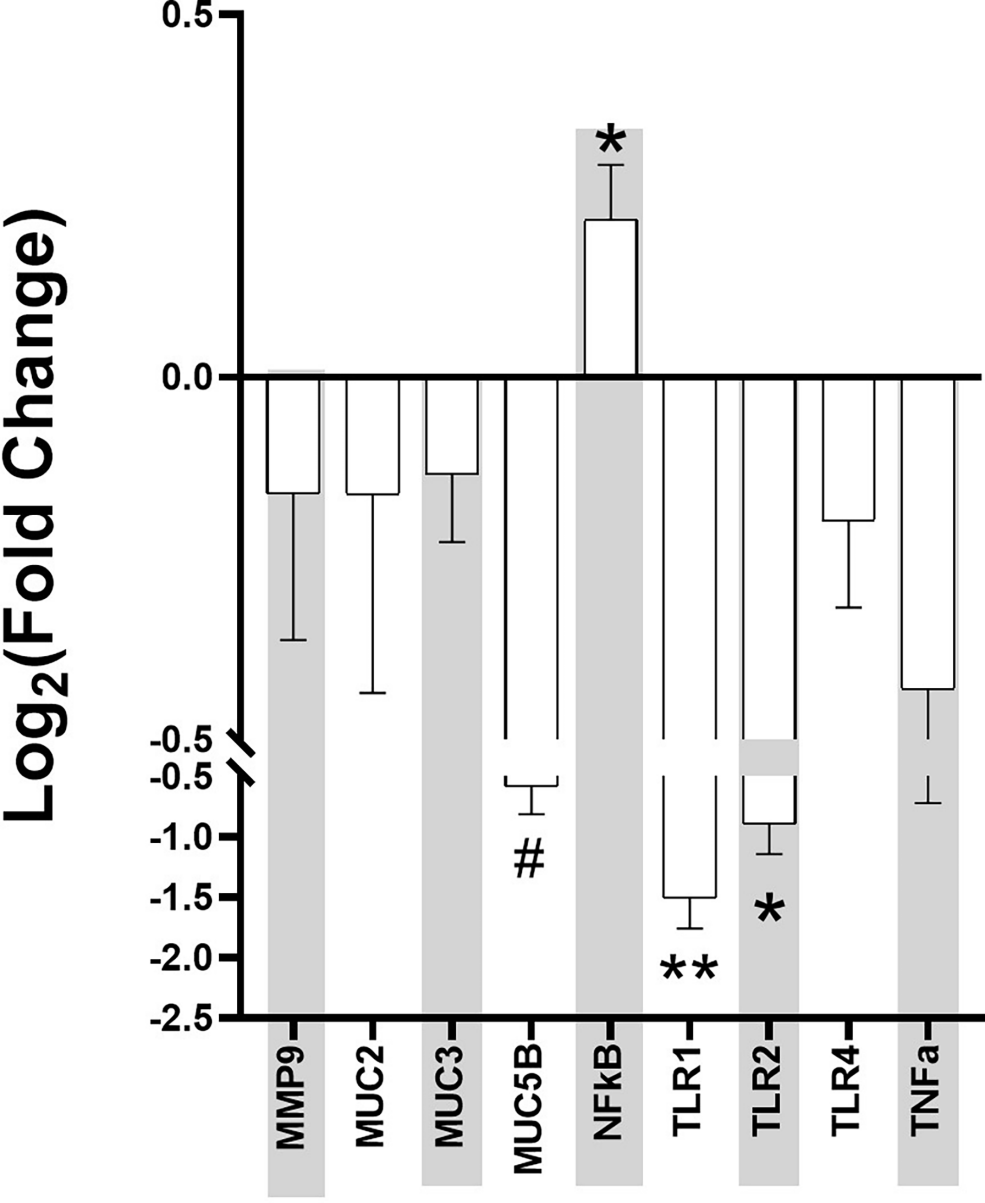
Intestinal Permeability Markers Fold Change at Week 4 post-stress in Aggressor-exposed social stress ileal samples. The qPCR for intestinal permeability markers were normalized by the housekeeping genes, and fold change of each Agg-E SS sample expression relative to the expression in healthy controls was calculated by the 2^(-ΔΔCt) method. Error bars are mean with the SEM (Standard Error of Mean). (#p-value 0.1<#<0.05, *p-value<0.05, **p-value<0.01).

Ileum samples from the mice were collected at Week 4 post-stress and used for RT-PCR using genes related to intestinal permeability markers. The data was normalized by the three housekeeping genes of *gapdh, actb, and ubc*, and the Log_2_ fold change between the control and Agg-E SS mice was calculated (**Figure 5**). The intestinal permeability markers of *muc5b* (p = 0.05), *nfkb* (p = 0.04), *tlr1* (p < 0.01), and *tlr2* (p = 0.04) were significant.

## 4 Discussion

The longitudinal dynamics, physiological effects, and the magnitude of the gut microbiome in response to stress is poorly understood. Our previous study (Gautam et al., 2018) explored the acute effects of PTSD on the gut microbiome, through the same 10-day experimental setup of mice undergoing 6-hours/day SS. Expanding from this result, the current study aimed to probe the long-term effects of Agg-E SS. Our ultimate objective is to find potential mitigation strategies of PTSD by suggesting modifications of the microbiome through nutraceutical interventions. A model estimating the shift in microbial composition could be used as a diagnostic marker of the host stress response, offering precision treatment through regulation of the microbiome, without spending time and money on novel interventions. Potential nutraceutical interventions may include prebiotics and probiotics consisting of special diets or dietary supplements, or postbiotics, which may include molecules that are able to influence the gut microbiome signals (Osadchiy et al., 2019).

There was a temporal change in the microbiome between control and Agg-E SS mice, as evidenced by the significant Chao1 alpha diversity metric. The Chao1 Index decreased between Baseline and Day 6 in both the Agg-E SS and control mice, but then increased, which could be attributed to the 6-hours/day of food and water deprivation. The beta diversity Weighted UniFrac PCoA showed evidence of change based upon both time and social stress. Incorporating the alpha and beta diversity is important in evaluating the microbial community over time (Wagner et al., 2018). In this study, SS is the driving force between the cohorts, but SS in alpha diversity was insignificant. Alpha diversity metrics capture the within sample changes but do not express changes in the community composition, as a community can undergo a complete compositional shift over time, but still have similar alpha diversity metrics (Wagner et al., 2018). These results seem to suggest that in Agg-E SS, there is a driving force that is reflected as a between communities shift, since comprehensive changes were seen between Agg-E SS and controls, thus giving insight into the severity and uniqueness of PTSD. A number of other studies corroborate our findings of no significant changes in alpha diversity, in PTSD in humans (Hemmings et al., 2017), in humans with psychological distress (Peter et al., 2018), and in our previous study of Agg-E SS mice (Gautam et al., 2018).

The phyla *Verrucomicrobia* and *Actinobacteria* experienced social stress-related changes, especially at the post-SS period where significant changes were observed at Week 1 and Week 4 post-SS. This showed a trend in the changing microbial populations due to delayed effects of Agg-E SS. Another study found that decreased abundance of *Actinobacteria*, *Lentisphaerae*, and *Verrucomicrobia* was associated with PTSD status in a human study of South African PTSD-affected individuals and trauma-exposed controls (Hemmings et al., 2017). In the present study, our Agg-E SS mice had increased *Actinobacteria*, but decreased *Verrucomicrobia*. We previously reported that *Verrucomicrobia*, *Actinobacteria*, and *Proteobacteria* were among the top enriched phyla during the 10-day study of Agg-E SS (Gautam et al., 2018). Our previous study also showed an immediate and persistent decrease in *Verrucomicrobia* over the ten days of SS (Gautam et al., 2018), and our current study had a decrease in *Verrucomicrobia* in Agg-E SS mice, with significance as compared to controls at the post-SS time points.

In regard to the rank abundance profile of the top genera, our previous study found *Akkermansia* to be the top genera in Agg-E SS mice (Gautam et al., 2018), as did the current study. *Akkermansia* is a genus in the phylum *Verrucomicrobia* and has had anti-inflammatory effects in a mouse model of chronic colitis (Zhai et al., 2019). *A. muciniphila* is a mucus colonizer and it uses mucin as its sole carbon and nitrogen source, but there is conflicting evidence of the beneficial nature of *Akkermansia* in Irritable Bowel Disease (IBD), as several studies found *Akkermansia* to be decreased or unaltered in IBD, while there is evidence it is increased in mice with colitis (Zhai et al., 2019).

In addition, *Firmicutes* and *Bacteroidetes* are the most abundant members of the mouse gut microbiome, and the ratio can change over time, in response to different factors. SS effected the *Firmicutes*/*Bacteroidetes* Log_10_ ratio although the difference between the phyla individually were not significant at any specific time points, but *Firmicutes* was significantly affected by time. In our previous manuscript (Hammamieh et al., 2012), we found that the average body weight increased overall, and the Agg-E SS mice gained significantly more weight during the 10-day social stress. Control mice decreased in weight initially after Day 4 of the 6-hours/day food and water deprivation, but the Agg-E SS mice weight steadily and significantly increased during the 10-day social stress. After 2 weeks of post-stress rest, the weight difference between control and Agg-E SS mice was no longer significant. Hormones such as leptin and ghrelin are involved in the process of food intake, weight, and energy balance, as leptin suppresses food intake and induces weight loss whereas ghrelin has a role in initiating food intake (Klok et al., 2007). An increase in *Firmicutes* and the *Firmicutes*/*Bacteroidetes* ratio is associated with weight gain, and the change in the *Firmicutes*/*Bacteroidetes* ratio may be accounted for by social stress, which is differentiating the two groups and affecting the microbiome (Gautam et al., 2018).

PTSD may affect the gut-brain axis, causing dysbiosis through psychological and physiological stress. In a previous study (Gautam et al., 2015), we found that 24hrs after Day 10 of social stress, there were significant changes in the plasma proteins of Agg-E SS mice, including haptoglobin, myeloperioxidase, and serum amyloid P-component that are known to be affected by inflammation, as well as mRNA data showing signs of liver inflammation 24hrs post-stress exposure. In addition, we previously found activation of inflammatory pathways in the hemibrain, blood, and spleen observed ten days and 42 days post-SS [10], and plasma protein levels of gut-derived metabolites were modified 24-hours post-SS and remained altered 4-weeks post-SS (Gautam et al., 2015). In the gastrointestinal tract, inflammation may lead to intestinal permeability (Maes et al., 2001; Klindworth et al., 2013). The current study looked into known markers for intestinal permeability in ileum samples collected at Week 4 post-SS, and the Log_2_ fold change of *muc5b*, *nfkb*, *tlr1*, and *tlr2* were significant when comparing Agg-E SS mice to the control mice. The other markers of intestinal permeability that were not significant between Agg-E SS and control mice may be due to the fact that the ileum samples were collected at Week 4 post-stress, and their effects may have been more pronounced during stress or earlier during post-stress. For example, *mmp-9* is a protease known to degrade extracellular matrix components, is increased in response to stress and may cause inflammation (Aguayo et al., 2018; Al-Sadi et al., 2019; Martinelli et al., 2021), and increased levels of *mmp-9* cause increased intestinal permeability (Al-Sadi et al., 2019). We had previously found that *mmp-9* was elevated in the blood directly after 5-days and 10-days of social stress (Muhie et al., 2017), but here it was down-regulated in the ileum samples at Week 4 post-stress.

The mucin genes of *muc2*, *muc3*, and *muc5b* were all down-regulated in Agg-E SS mice and are involved in preserving the mucus layer that covers the gastrointestinal tract, which is the first line of defense against the contents of the lumen (Gaudier et al., 2004). The mucus layer pays a major protective role, and intestinal permeability may increase through stress by the decrease in mucus production (Soderholm et al., 2002; Barreau et al., 2004). Therefore, at Week 4 post-SS the mucin genes are down-regulated, which may point to a compromise in the intestinal barrier. *A. muciniphila*, a known mucin-degrading bacteria that utilizes mucin as its sole carbon and nitrogen source, is increased in Agg-E SS mice, and may be affecting the mucin gene expression.

Toll-like receptor (TLR) signaling in the gut is involved in maintaining homeostasis, however, under inflammatory conditions, the mucosal epithelial TLR expression is increased which contributes to both the induction of the inflammatory response and immune tolerance (Sapone et al., 2011). The Log_2_ fold change of the TLR genes of *tlr1*, *tlr2*, and *tlr4* were all down-regulated in the Week 4 ileum samples in Agg-E SS mice. Due to using Week 4 post-stress ileum samples, we may have overlooked earlier activation of the TLR genes in the ileum of Agg-E SS mice. Our previous manuscript regarding Agg-E SS on brain transcriptomics, found that *tlr1* was predicted to be up-regulated in the hippocampus immediately following 5 days of SS and both *tlr1* and *tlr4* were predicted to be down-regulated in the septal region and amygdala, respectively, at 6 weeks post-10 day SS (Muhie et al., 2015). Another of our previous studies (Gautam et al., 2015) found Lipopolysaccharide (LPS) to be to topmost activator in Agg-E SS differentially expressed genes in the liver at 24 hours-post stress, but it trended towards being inhibited at 1.5 weeks and 4 weeks post-stress. LPS is a ligand of TLR activation, and may lead to the activation of *nfkb* gene expression (Savinova et al., 2009). We did find that *nfκb* was significantly up-regulated in Agg-E SS mice. The transcription factor *nfκb* plays a key role in immunity and inflammation and is damaging agent that is part of potential mechanisms of oxidant-induced intestinal barrier disruption. In our previous study, at later time points of 10-days post-stress and 6 weeks post-stress, pathways related to *nfkb*-regulated transcriptions were activated in the blood, brain, and spleen (Muhie et al., 2017). *nfκb1* was up-regulated in the corpus striatum after ten-days SS followed by 24 hours of rest, and in the amygdala *nfκb1* was down-regulated and *nfκb2* was up-regulated after ten-days SS followed by 42 days post-stress rest (Muhie et al., 2015). *Nfκb1* and *Nfκb2* proteins p105 and p100 serve both as *nfkb* precursors and inhibitors of *nfkb* dimers (Savinova et al., 2009). The binding of *nfkb* to the DNA promoter region in the nucleus leads to up- or down-regulation, and *nfkb* was shown to regulate *tnf-alpha*, which led to increased intestinal permeability (Ma et al., 2004).

Pathway analysis of predicted MetaCyc pathways showed that all of the pathways significant between AggE-SS and control mice were involved in biosynthesis and degradation/utilization/assimilation superclasses, which are complimentary processes of bioenergetic networks. Biosynthesis comprises the pathways involved in the full spectrum of the biosynthetic capabilities of cells that stimulate their growth and interactions, and degradation/utilization/assimilation contains the pathways by which various organisms degrade substrates to serve as energy and nutrients. Human PTSD patients have a known energy imbalance, as evidenced in studies by a lower mitochondrial DNA copy number in PTSD, which may reflect impaired energy metabolism and represent a novel aspect of PTSD pathophysiology (Bersani et al., 2016; Mellon et al., 2018; Bersani et al., 2020). Metabolic profiling identified significant differences between PTSD positive and negative humans in the biochemical pathways involved in glucose metabolism, energy utilization, and lipid metabolism (Mellon et al., 2019), further illustrating the energy needs in PTSD. We had previously found that in our Agg-E SS mouse model, lipid metabolites were elevated in plasma at 24hrs, 1.5 weeks, and 4 weeks post-stress, which may indicate increased fat utilization due to increased energy needs during and after Agg-E SS (Gautam et al., 2015). Therefore, PTSD may cause an increased energy utilization, which leads to the need for more energy. As the energy needs increase in the Agg-E SS mice, they may start using energy salvage pathways and non-conventional energy networks to overcome their energy needs. The pathways of 1,4-dihydroxy-6-naphthoate biosynthesis I and 1,4-dihydroxy-6-naphthoate biosynthesis II were predicted to be up-regulated at Day 6, Day 10, Week 1, and Week 4 in Agg-E SS mice and are alternate pathways of methaquionone synthesis *via* futalosine, which is an important component of the electron-transfer system in prokaryotes (Seto et al., 2008; Zhi et al., 2014). Also, the Superpathway of menaquinol-8 biosynthesis II was predicted to be up-regulated at these time points, and is the well-characterized route to synthesize menaquinone from chorismate (Hiratsuka et al., 2008). Day 10 of acute stress had a predicted activation in Agg-E SS mice of the Superpathway of glycerol degradation to 1,3-propanediol, which is an alternate pathway for glycerol and occurs in the absence of an external oxidant, thus glycerol is fermented by a dismutation process (Rush et al., 1957). Week 1 had a predicted up-regulation of the Superpathway of thiamin diphosphate biosynthesis II and the component pathway of thiazole biosynthesis II (*Bacillus*); both are involved in the synthesis of the thiazole complex of thiamin, which is part of thiamin diphosphate (vitamin B1) synthesis (Lawhorn et al., 2004). Week 1 had a predicted inhibition of Starch Degradation V, which is an alternate pathway where starch is degraded extracellularly by amylopullulanase (Lee et al., 2006; Labes and Schönheit, 2007). Therefore, the pathway analysis identified a potentially perturbed cluster of bioenergetic networks, which became increasingly enriched with the time since the termination of Agg-E SS.

In addition to building upon our previous microbiome study (Gautam et al., 2018) by exploring the long-term effects of the 10-day Agg-E SS on the gut microbiota, we also sought to validate the findings from our previous experiment in an effort to identify sustained alterations in the fecal microbiome that need to be examined further. Regarding sample diversity, both studies showed no significant change in the alpha diversity metrics of the Shannon and Simpson’s Indexes, and time and SS influenced beta diversity. The phyla most highly ranked in abundance were *Firmicute*s and *Bacteroidetes*, followed by *Tenericutes*, *Verrucomicrobia*, *Actinobacteria* and *Proteobacteria* in the previous study, whereas the current study had the same order of phyla abundance, except *Tenericutes* followed *Proteobacteria* in rank. In addition, the phyla *Verrucomicrobia* and *Actinobacteria* were significantly different between control and Agg-E SS mice and the *Firmicutes/Bacteroidetes* ratio was affected by SS. Furthermore, *Akkermansia* was identified to be the top genera in Agg-E SS mice in both studies. This study focused on long-term effects following stress and used data from the pre-stress, post-stress and recovery time points.

## 5 Conclusion

The current longitudinal study illustrated that exposure of mice to social conflicts cause lasting shift in the gut microbiome. Hence, microbial signatures could be valuable tools in managing long-term impacts of social stress. The ultimate objective is to identify potential mitigation strategies for PTSD. The alpha and beta diversity data, as well as the taxonomy alterations illustrated that PTSD-like Agg-E SS is making comprehensive changes between communities, which indicates that therapeutic, nutraceutical interventions (prebiotics, probiotics, and postbiotics) could be a viable solution, since there are distinct differences between control and SS mice. Regarding the gut microbiome, our understanding of the complex processes at work is still lacking, and more work needs to be done to elevate our comprehension. In addition, this study involved 16S rRNA sequencing, which is more limited than in-depth whole genome shotgun sequencing at identifying bacteria at the species level of classification, as well as identifying the complete microbial composition to include the other kingdoms of viruses and fungi. Furthermore, this study only looked at feces and did not take into account the rest of the gut microbiome over the length of the intestinal tract nor the mucosal population. In addition, this study stopped at Week 4 post-SS, and examining the microbiome further post-stress would be beneficial, to examine the lasting impacts or complete recovery and stabilization of the microbiome post-SS. It is important to characterize the fecal, luminal, and mucosal populations and their relationships to attain the complete picture. Mouse ecology does vary from humans, so there is a need to test in phylogenetically higher orders of animals or human, to gain more confidence in the results. Nevertheless, this study does illustrate the effect of life-changing trauma simulating PTSD does have a long-term effects on the microbial community, as we did observe changes at Week 4 post-stress in a mouse model, which equates to approximately two human years.

## Data Availability Statement

The sequence data generated and analyzed in this study were deposited in the NCBI Sequence Read Archive under Bioproject PRJNA769427.

## Ethics Statement

The animal study was reviewed and approved by The Institutional Animal Care and Use Committee (IAUCUC) at the US Army Center for Environmental Health (USACEHR).

## Author Contributions

AH wrote the manuscript. AG and NC conducted and coordinated experiments using the mouse model and obtained samples. AH analyzed the data. MJ and RH conceived the study and participated in the design. All authors contributed to the article and approved the submitted version.

## Funding

The support from USAMRMC grant number 09284002 is gratefully acknowledged.

## Author Disclaimer

Material has been reviewed by the Walter Reed Army Institute of Research. There is no objection to its presentation and/or publication. The opinions or assertions contained herein are the private views of the author, and are not to be construed as official, or as reflecting true views of the Department of the Army or the Department of Defense. Research was conducted under an approved animal use protocol in an AAALAC International accredited facility in compliance with the Animal Welfare Act and other federal statutes and regulations relating to animals and experiments involving animals and adheres to principles stated in the Guide for the Care and Use of Laboratory Animals, NRC Publication, 2011 edition.

## Conflict of Interest

The authors declare that the research was conducted in the absence of any commercial or financial relationships that could be construed as a potential conflict of interest.

## Publisher’s Note

All claims expressed in this article are solely those of the authors and do not necessarily represent those of their affiliated organizations, or those of the publisher, the editors and the reviewers. Any product that may be evaluated in this article, or claim that may be made by its manufacturer, is not guaranteed or endorsed by the publisher.

## Abbreviations

Agg-E, aggressor-exposed; ASVs, amplicon sequence variants; LEfSe, linear discriminant analysis effect size; MANOVA, multivariate analysis of variance; OTU, operational taxonomic unit; PCoA, principle coordinate analysis; PERMANOVA, permutational multivariate analysis of variance; PTSD, post-traumatic stress disorder; SEM, standard error of mean; SS, social stress.
