# Supplementary figures and images for: Acute and Delayed Effects of Stress Eliciting Post-Traumatic Stress-Like Disorder Differentially Alters Fecal Microbiota Composition in a Male Mouse Model
Source: Front Cell Infect Microbiol. 2022 Mar 1;12:810815. doi: 10.3389/fcimb.2022.810815 (PMC8921487; doi:10.3389/fcimb.2022.810815)

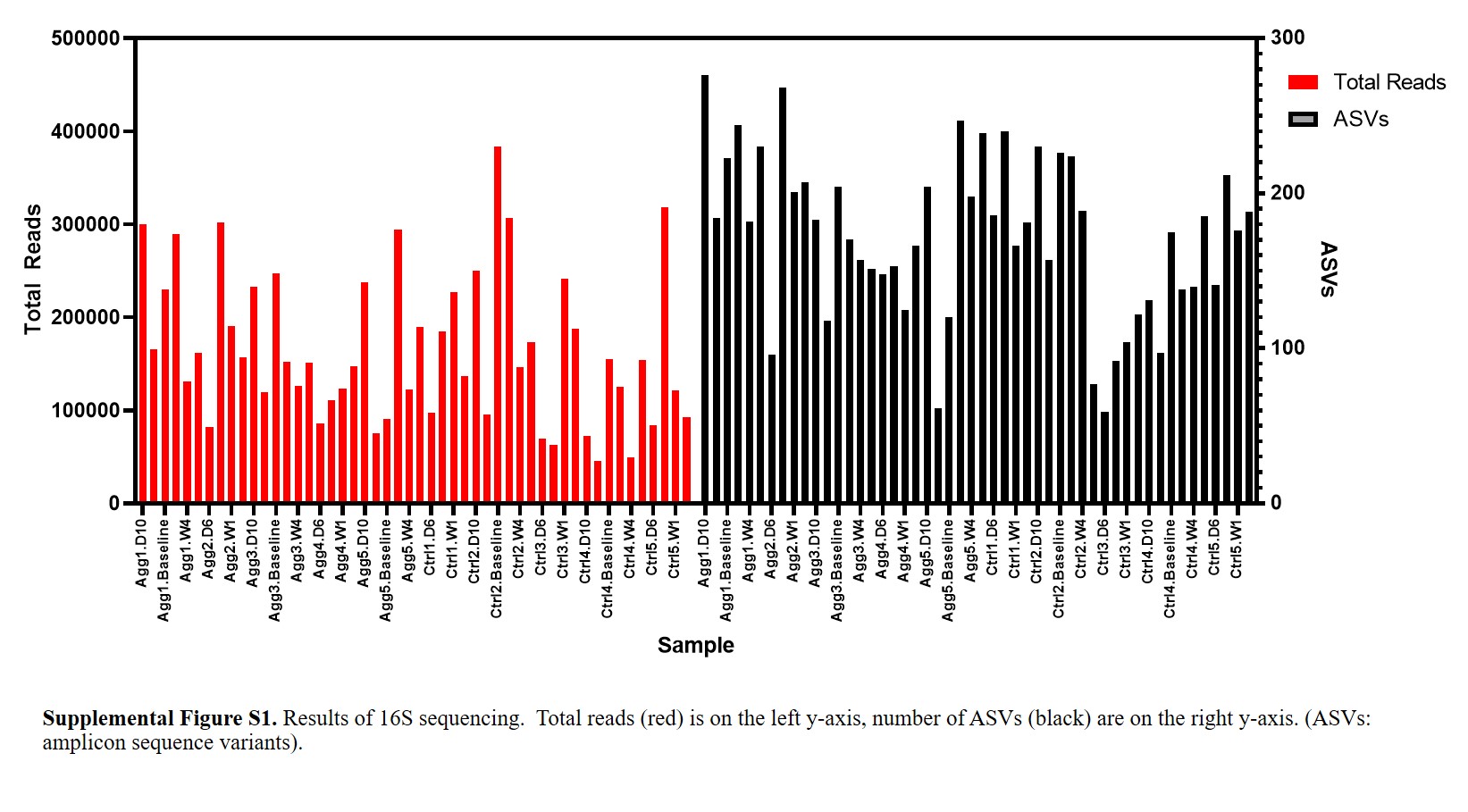

Supplement: Supplementary file 1 [file Image_1.jpeg]

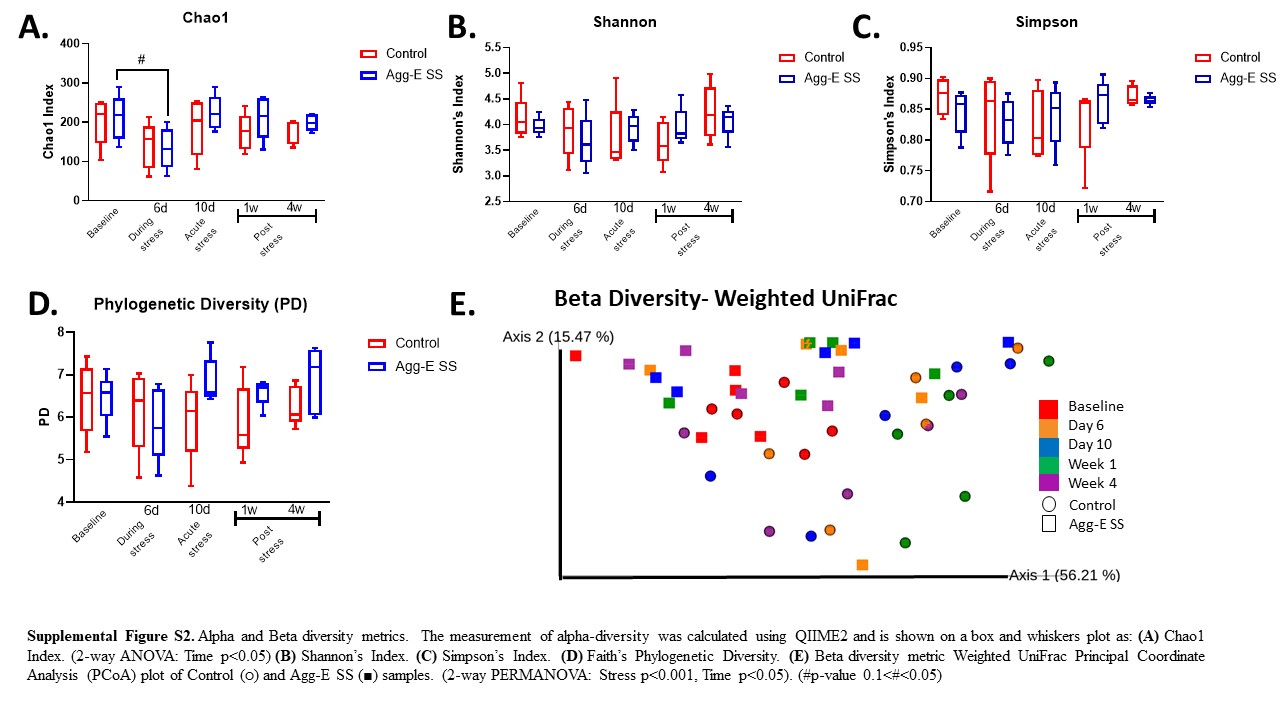

Supplement: Supplementary file 2 [file Image_2.jpeg]

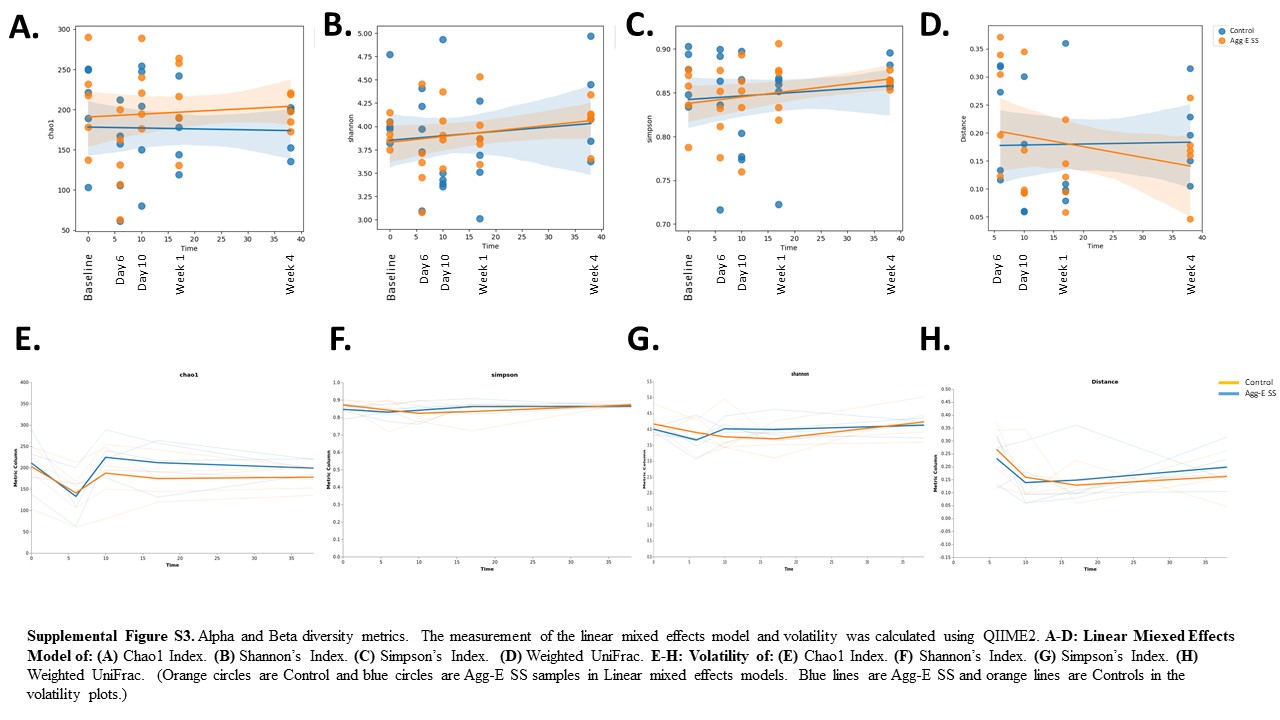

Supplement: Supplementary file 3 [file Image_3.jpeg]

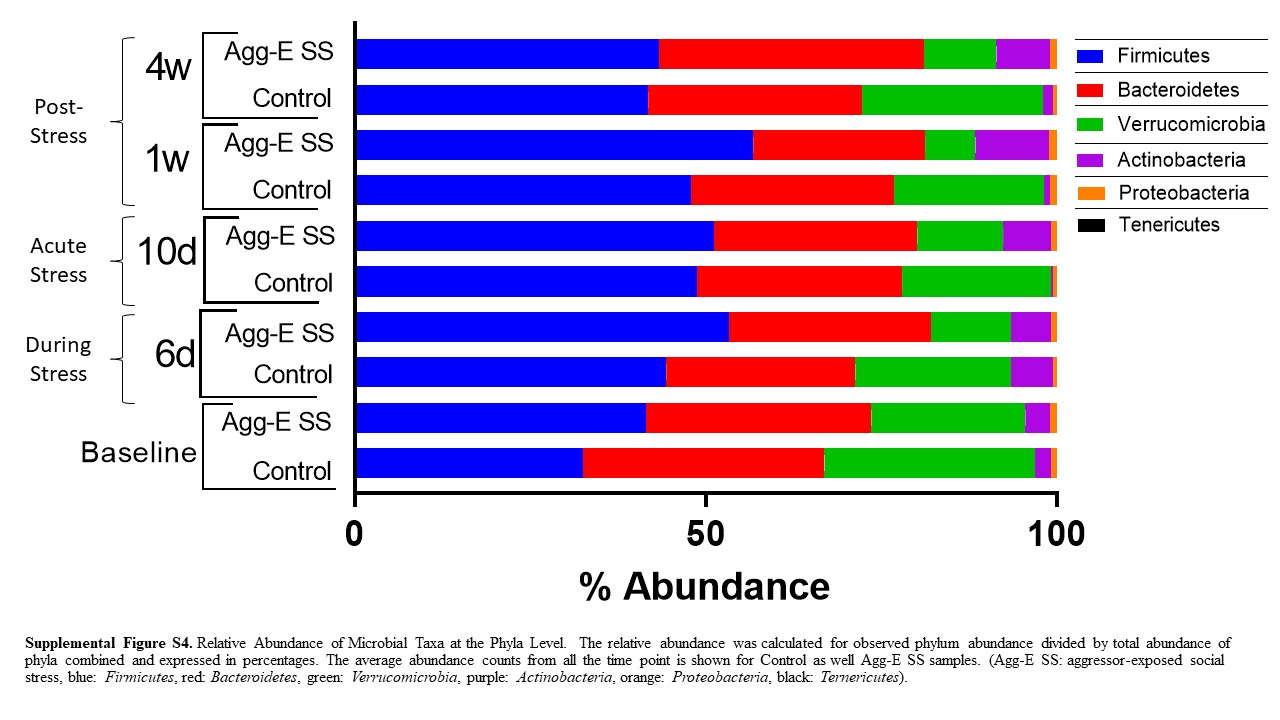

Supplement: Supplementary file 4 [file Image_4.jpeg]

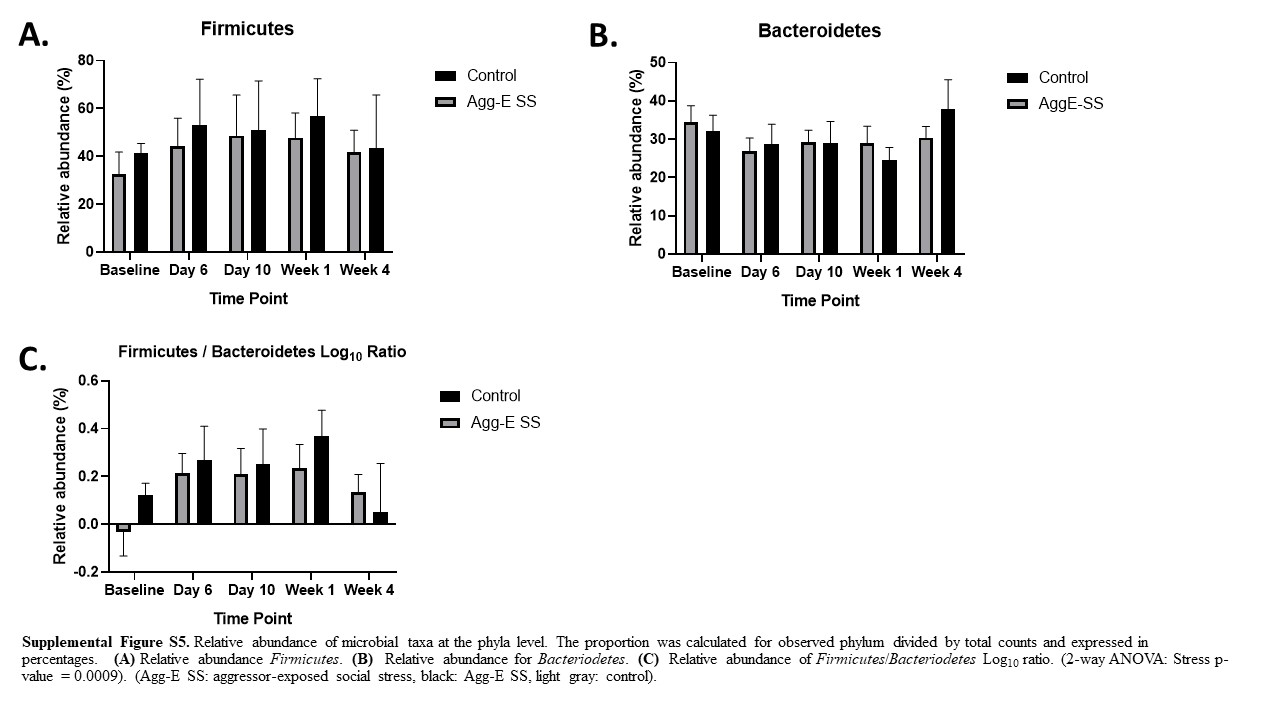

Supplement: Supplementary file 5 [file Image_5.jpeg]

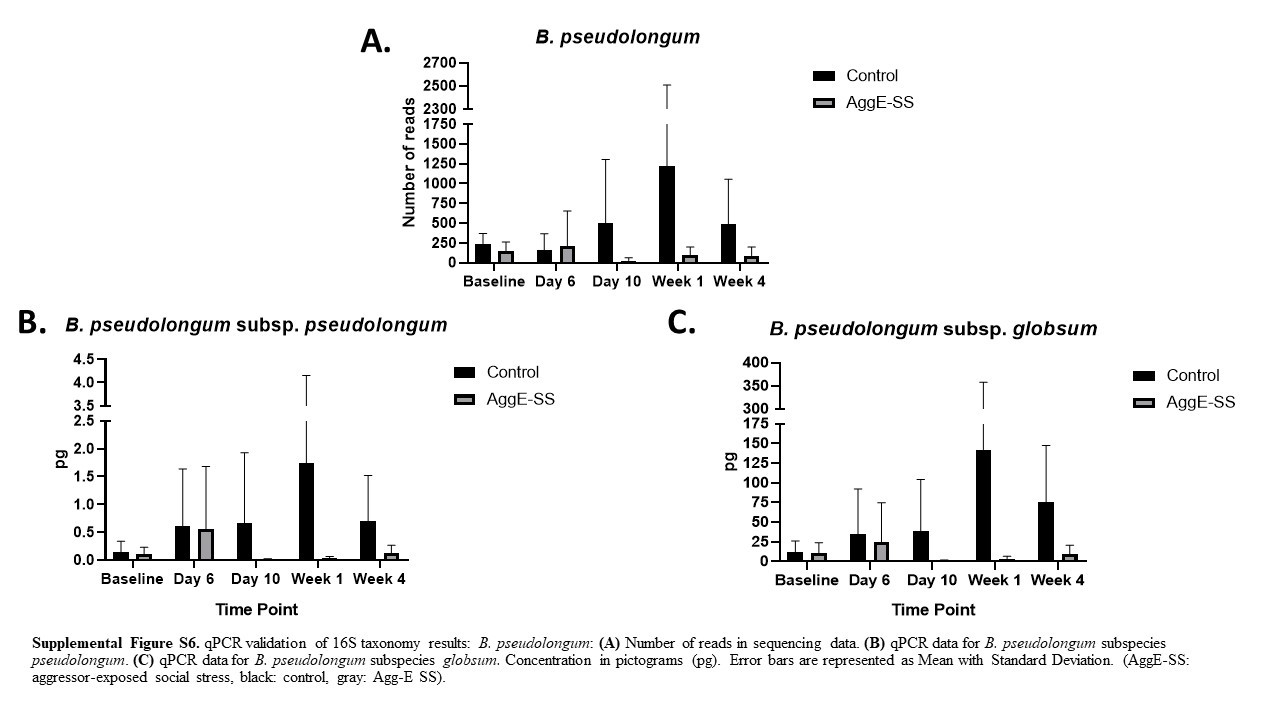

Supplement: Supplementary file 6 [file Image_6.jpeg]

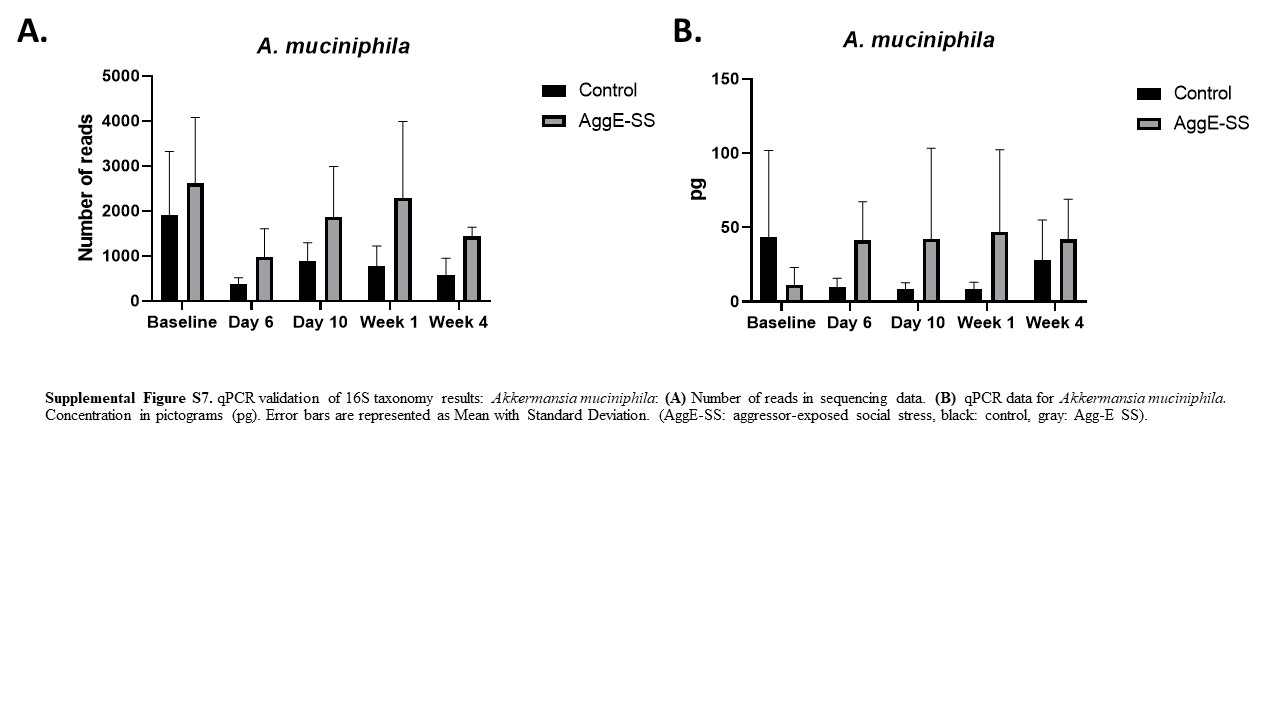

Supplement: Supplementary file 7 [file Image_7.jpeg]
